# Dynamics of Superparamagnetic Iron Oxide Nanoparticles with Various Polymeric Coatings

**DOI:** 10.3390/ma12111793

**Published:** 2019-06-03

**Authors:** Tomasz Strączek, Sylwia Fiejdasz, Damian Rybicki, Kamil Goc, Janusz Przewoźnik, Weronika Mazur, Maria Nowakowska, Szczepan Zapotoczny, Stanisław Rumian, Czesław Kapusta

**Affiliations:** 1 Department of Solid State Physics, Faculty of Physics and Applied Computer Science, AGH University of Science and Technology, Mickiewicza 30, 30-059 Krakow, Poland; ryba@agh.edu.pl (D.R.); Kamil.Goc@fis.agh.edu.pl (K.G.); januszp@agh.edu.pl (J.P.); weronika1734@gmail.com (W.M.); kapusta@agh.edu.pl (C.K.); 2 Faculty of Chemistry, Jagiellonian University, Gronostajowa 2, 30-387 Kraków, Poland; nowakows@chemia.uj.edu.pl (M.N.); zapotocz@chemia.uj.edu.pl (S.Z.); 3 The Interuniversity Center for The New Techniques and Medical Technologies, Cracow University of Technology, Warszawska 24, 31-155 Kraków, Poland; starum@op.pl

**Keywords:** iron oxide nanoparticles, superparamagnetism, polymer coatings, Mössbauer spectroscopy, alternating current magnetic susceptometry (ACMS), magnetic dynamics

## Abstract

In this article, the results of a study of the magnetic dynamics of superparamagnetic iron oxide nanoparticles (SPIONs) with chitosan and polyethylene glycol (PEG) coatings are reported. The materials were prepared by the co-precipitation method and characterized by X-ray diffraction, dynamic light scattering and scanning transmission electron microscopy. It was shown that the cores contain maghemite, and their hydrodynamic diameters vary from 49 nm for PEG-coated to 200 nm for chitosan-coated particles. The magnetic dynamics of the nanoparticles in terms of the function of temperature was studied with magnetic susceptometry and Mössbauer spectroscopy. Their superparamagnetic fluctuations frequencies, determined from the fits of Mössbauer spectra, range from tens to hundreds of megahertz at room temperature and mostly decrease in the applied magnetic field. For water suspensions of nanoparticles, maxima are observed in the absorption part of magnetic susceptibility and they shift to higher temperatures with increasing excitation frequency. A step-like decrease of the susceptibility occurs at freezing, and from that, the Brown’s and Néel’s contributions are extracted and compared for nanoparticles differing in core sizes and types of coating. The results are analyzed and discussed with respect to the tailoring of the dynamic properties of these nanoparticle materials for requirements related to the characteristic frequency ranges of MRI and electromagnetic field hyperthermia.

## 1. Introduction

Recently, nanotechnology has been receiving a great deal of interest in many branches of science and technology, especially in medicine and biotechnology [1]. Advances in the biomedical sector are strongly influenced by nanoparticle research due to a wide range of possible applications; e.g., in drug delivery [2,3], cancer detection [4], tissue regeneration [5], magnetic resonance imaging (MRI) [6,7] or magnetic fluid hyperthermia (MFH) [8,9]. Among the various types of nanoparticles, those with magnetic functionality are attracting a great deal of interest. Superparamagnetic iron oxide nanoparticles (SPIONs) and, in particular, magnetite (Fe_3_O_4_) and maghemite (γ-Fe_2_O_3_)-based nanoparticles, exhibit relevant characteristics of remarkable magnetic properties and an appropriate size and biodegradability, which make them attractive for various medical applications [10].

The magnetic properties of iron oxide nanoparticles are strongly dependent on their composition and morphology. That is why selecting the proper preparation method is crucial for the further success of the planned application. There are several different approaches to choose from; however, chemical methods are most often employed, since in the case of physical methods—e.g., powder ball milling—there is a problem with controlling the size of nanoparticles [11]. The most commonly used synthetic routes are thermal decomposition [12], sol-gel [13], microemulsion [14], microwave-assisted and hydrothermal reactions [15]. Among them, the co-precipitation method is particularly attractive since it is easy, fast and efficient. In order to prevent aggregation and to increase biocompatibility, iron oxide nanoparticles are usually coated during or after preparation. Polymers are most often employed as coating materials [16]. Synthetic polymers such as polyethylene glycol (PEG) [17], polyvinylpyrrolidone (PVP) [18], poly(lactic-co-glycolic acid) (PLGA) [19] and those of natural origin such as dextran [20], starch [21], chitosan [22], gelatin [23] and alginate [24] have been used in recent years as coatings for SPIONs. Other examples are fatty acids (oleic acid) [25], amino acids (arginine) [26], oxides (silica) [27,28] and metals (gold) [29]. Many different techniques have been used for the characterization of SPIONs. Core size and morphology can be studied by transmission electron microscopy (TEM) or atomic force microscopy (AFM) [30]. X-ray diffraction (XRD) and, to some extent, Mössbauer spectroscopy (MS) can provide information on the crystal structure and size of crystallites [31]. The hydrodynamic diameter can be obtained using the dynamic light scattering (DLS) method [32]. DLS also provides information concerning the colloidal stability and aggregation of SPIONs. Using nuclear magnetic resonance (NMR) spectroscopy/relaxometry, the *T*_1_, *T*_2_ proton relaxation times can be measured [33]. Magnetic properties are usually studied using a vibrating sample magnetometer (VSM) or superconducting quantum interference device (SQUID) [34,35].

Regarding the potential biomedical applications of nanoparticles (e.g., in MRI, MFH), the dynamics of SPIONs is also very important. Mössbauer spectroscopy and alternating current magnetic susceptometry (ACMS) are tools which can give a deeper insight into their dynamic behavior.

SPIONs have been intensively studied for their theranostic purposes, aiming to combine diagnoses with therapeutic effects. The visualization of tumors and inflammatory lesions serves as an example of their usefulness in diagnostic process. Because SPIONs can shorten proton relaxation times *T*_2_ and *T*_2_^*^, they have a great potential for MRI as so-called negative contrast agents [36,37]. SPIONs with a modified surface and attached monoclonal antibodies were proposed as contrast agents for MRI at the early stage of endothelial inflammation [38]. SPIONs coated with PLGA have been also used for in vivo MRI [39].

Magnetic fluid hyperthermia can be applied in tumor therapy. In that method, SPIONs are exposed to an alternating external magnetic field that triggers the heating and/or motion of particles, causing local heating and leading to the death of tumor cells. SPIONs functionalized by curcumin conjugate were shown to be promising anti-cancer agents, combining the hyperthermal effect and drug carrier functionality [40]. Functionalized superparamagnetic iron oxide nanoparticles were proposed to treat liver cancer via MFH-based thermotherapy [41].

This paper presents a thorough study into SPION materials, including the preparation and characterization of their crystal structure, composition, size of nanoparticles, magnetic properties and dynamics in view of their possible application as contrast agents in MRI and electromagnetic power absorbing media in hyperthermia therapy.

## 2. Materials and Methods

### 2.1. Materials

**Scheme 1:**
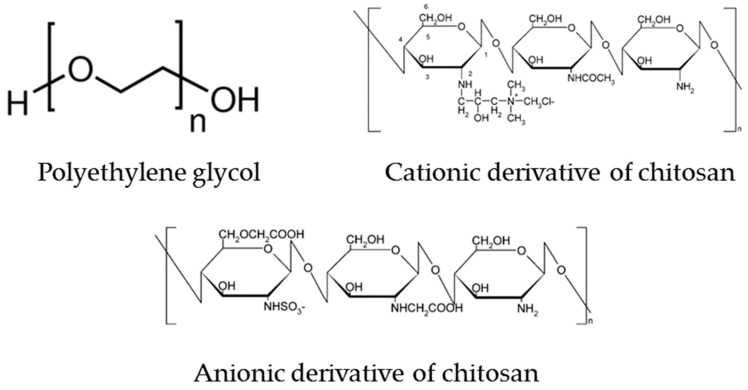
Polymers used as coatings of iron oxide nanoparticles.

Iron(III) chloride hexahydrate and iron(II) chloride tetrahydrate (Sigma-Aldrich, St. Louis, MO, USA), ammonia (25% solution, puriss. p.a) (Sigma), diethylenetriaminepentaacetic acid gadolinium(III) dihydrogen salt hydrate 97% (GdDTPA, Sigma-Aldrich), chitosan (low molecular weight, Aldrich), and mili-q water to prepare solutions, and iron oxide nanoparticles with PEG at a size of 30 nm (Ocean NanoTech, LCC, San Diego, CA, USA) were used. Cationic (CCh) and anionic (ACh) derivatives of low molecular weight chitosan were obtained according to the procedures described previously [42]. The modification degree for CCh (DS_GTMAC_) was found to be of 57%, and in the case of ACh (DS_TMST_), it was equal to 67%. The structures of polymer coatings of SPIONs (Scheme 1) are presented below.

Also, microcrystalline magnetite and maghemite powders (Sigma) were used in the study as reference materials.

### 2.2. Preparation of Iron Oxide-Based Nanoparticles

Superparamagnetic iron oxide nanoparticles coated with a cationic derivative of chitosan (SPION-CCh) were obtained using the procedure developed by us and described earlier [43]. Briefly, iron salts in a molar ratio Fe(III):Fe(II) = 2:1 were dissolved in 50 mL of aqueous solution of CCh (1 g/L). The amounts of iron oxides used for the synthesis of all the samples were 0.1622 g of FeCl_3_·6H_2_O and 0.0596 g of FeCl_2_·4H_2_O. The solution was deoxygenated by purging with argon and sonicated for 10 min in a thermostated bath (20 °C). Next, 5 mL of 5 M NH_3_ (aq) was added drop-wise, and the solution was further sonicated for 30 min. Finally, the precipitated nanoparticles were purified by magnetic filtration. SPION-CCh-B was prepared using the analogous procedure as in the case of SPION-CCh but without sonication and argon bubbling. The final product was obtained by magnetic separation. SPIONs with a surface-attached gadolinium complex SPION-Gd (ACh-GdDTPA) were obtained using the procedure developed by us and described previously [44]. Briefly, in the first step, the ACh polymer was modified with gadolinium complex GdDTPA. In the next step, SPION-CCh was coated with ACh-GdDTPA.

### 2.3. Experimental

#### 2.3.1. Dynamic Light Scattering (DLS)

Hydrodynamic sizes and zeta potentials of the obtained nanoparticles were measured by DLS using ZetaSizerNano ZS (Malvern Instruments Ltd., Royston, UK), equipped with a He-Ne laser operating at 633 nm wavelength. The measurements were performed at 25 °C, three times for each sample. The weighted size according to distribution by number, as well as zeta potential, were determined.

#### 2.3.2. Scanning Transmission Electron Microscopy (STEM)

The size and shape of nanoparticles studied were determined by STEM with the FEI Nova NanoSEM 450 apparatus (Waltham, MA, USA). An aqueous dispersion of nanoparticles was deposited on a carbon-coated copper mesh grid and air-dried at room temperature.

#### 2.3.3. Alternating Current Magnetic Susceptibility (ACMS)

To obtain detailed information regarding magnetic dynamics, the ACMS method was employed. ACMS is a method of measuring the magnetic moment of a sample induced with an alternating field produced by a coil. The resultant voltage induced at the receiving coil is measured and allows for the determination of the “in phase” (dispersion) and “out of phase” (absorption) parts of the magnetic susceptibility. ACMS is particularly sensitive to magnetic phase transitions; therefore, it is very useful in studies of superparamagnetism.

#### 2.3.4. Mössbauer Spectroscopy (MS)

The method exploits the recoilless absorption of gamma rays produced by nuclear transition. From the hyperfine parameters derived from the spectrum, the information on the electronic and magnetic state of the Mössbauer element in the material can be obtained. In particular, the isomer shift (*IS*) provides the information on the electron density at the nucleus, the quadrupole splitting (*QS*) is a measure of the asymmetry of the electron charge distribution and the hyperfine field induction (*B_HF_*)—i.e., the magnetic field at the nucleus—can provide information on the magnetic moment of the atoms. ^57^Fe Mössbauer measurements have been carried out in the transmission mode with a constant acceleration spectrometer. A source of 50 mCi ^57^Co in a rhodium matrix has been used. For low-temperature measurements, a cold finger cryostat filled with liquid nitrogen or helium was used. An external magnetic field was produced by a set of neodymium permanent magnets.

The spectra were fitted using Gauss–Newton’s iterative method of minimizing χ^2^ with the Lorentz function shape of the spectral lines. For room-temperature results, a model proposed by Blume and Tjon was used to obtain relaxational parameters *F* (fluctuation frequency) and *ρ* (asymmetry as defined by D. G. Rancour) [45,46].

#### 2.3.5. X-Ray Diffraction (XRD)

The study of the structure and crystallite size was performed with X-ray diffraction. The experiments were performed with a Siemens D5000 diffractometer (Siemens, München, Germany) at room temperature. The Cu K_α_ source (wavelengths 1.540598 Å and 1.544426 Å) and a rear graphite monochromator were used. The samples were placed on a reflectionless silicon holder and fixed at the table of the goniometer. The 2*θ* ranged from 10° to 120° with a step of 0.03°.

## 3. Results and Discussion

### 3.1. Nanoparticle Materials Studied

Three types of iron oxide-based nanoparticles were prepared based on the method described previously by us for chitosan-coated SPIONs [43]. The positively charged SPIONs were obtained using the co-precipitation method of synthesis from the mixture of iron salts (FeCl_3_ and FeCl_2_) reacting with ammonia in the presence of a biocompatible polymer, cationic chitosan derivative (CCh). Cationic nanoparticles, named SPION-CCh-B, were also prepared using the co-precipitation method. In this case, the pulsed sonication, argon bubbling and thermostating at a temperature of 20 °C were not applied.

A portion of SPIONs was subjected to further modifications in order to introduce the gadolinium complex at their surfaces according to the procedure described earlier by us [44]. The modification method, giving a product marked as SPION-ACh-Gd, first involves the conjugation of gadolinium complex (GdDTPA) to the anionic derivative of chitosan. Then, that product was used for coating of the previously prepared SPION-CCh nanoparticles, utilizing the “layer-by-layer” (LbL) method.

The commercially available PEG-coated iron oxide nanoparticles of 30 nm in size (SPION-PEG Ocean NanoTech, LCC) were also used for comparison purposes in the current study.

### 3.2. Physicochemical Properties of the Materials Studied

#### 3.2.1. Results of DLS and Zeta Potential Measurements

DLS measurements were carried out to determine the size and zeta potential values of nanoparticle materials in water suspension. The values of the mean hydrodynamic diameter and the zeta potential are presented in Table 1. In the case of nanoparticles synthetized by us, for SPION-CCh and SPION-ACh-Gd, the hydrodynamic size was about 100 nm and the zeta potential values were in the range which ensured the electrostatic stabilization of their aqueous colloidal dispersions. The measurements revealed that the iron oxide-based sample termed SPION-CCh-B, which has a bigger hydrodynamic diameter of ca. 200 nm, possibly forms aggregates. That results from differences in the synthesis procedure and confirms the suggestion that sonication and argon bubbling considerably influence the size of the formed nanoparticles. For the commercially available iron oxide-based nanoparticles, SPION-PEG, the hydrodynamic diameter was found to be 49 nm, consistent with the value provided by the manufacturer.

The zeta potential values of the prepared nanoparticles (SPION-CCh and SPION-ACh-Gd) were in the range required for the formation of stable dispersions. That was not the case for commercial product (*ξ* = −9 mV for SPION-PEG). However, according to the manufacturer, even though these formulations have a zeta potential from −10 mV to 0 mV, these nanoparticles form a sterically stabilized aqueous colloidal dispersion.

#### 3.2.2. Results of STEM Study

**Figure 1:**
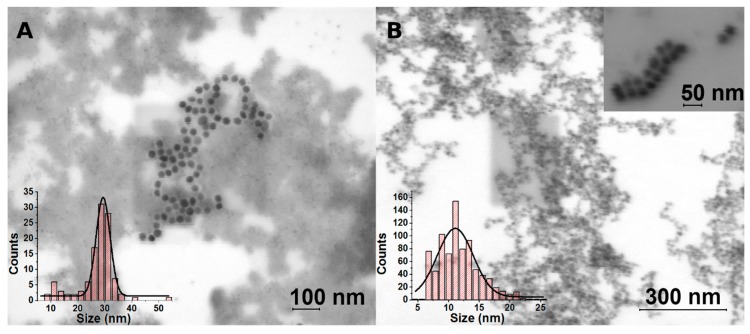
Bright-field scanning transmission electron microscopy (STEM) images: (**A**) SPION-PEG, (**B**) SPION-CCh.

The morphology of the synthesized and commercial nanoparticles was studied using STEM (Figure 1). By employing this technique, it was possible to directly observe the shape and size of the iron oxide cores. STEM images revealed that the cores are spherical and separated, confirming that they are covered with thin layers of polymers.

Using histograms obtained by ImageJ software (inset in Figure 1), the size of the nanoparticles studied was estimated. The cores of SPION-PEG were found to be of 30 nm, which is in agreement with the specification provided. In the case of SPION-CCh and SPION-ACh-Gd, the estimated values were about 10 nm and were consistent with those previously obtained from TEM measurements [43,44]. Unfortunately, it was not possible to precisely determine the size of SPION-CCh-B cores due to the strong tendency towards aggregation.

### 3.3. Structural and Magnetic Characterization

#### 3.3.1. Results of XRD Study

**Figure 2:**
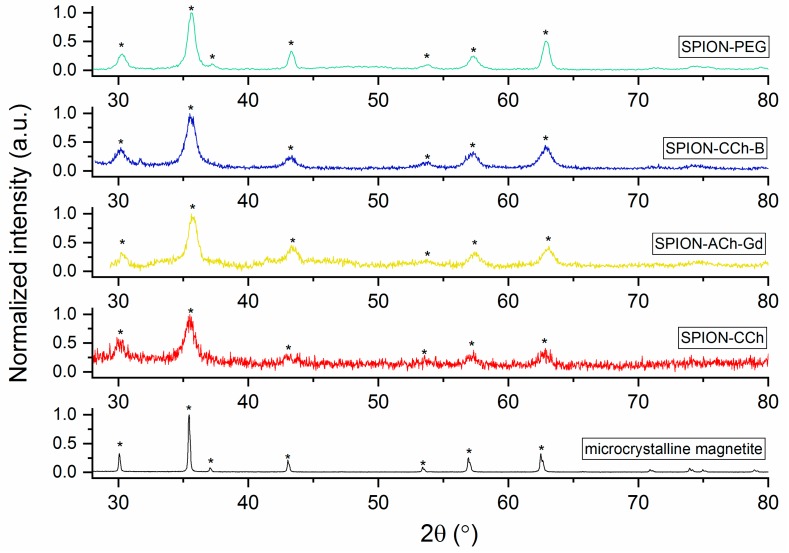
XRD patterns of microcrystalline magnetite, SPION-CCh, SPION-ACh-Gd, SPION-CCh-B and SPION-PEG.

The XRD patterns of microcrystalline magnetite, SPION-CCh, SPION-ACh-Gd, SPION-CCh-B and SPION-PEG are presented in Figure 2. Peaks at 2*θ* of 30°, 35°, 43°, 53°, 57° and 63° correspond to (220), (311), (400), (422), (511), (440), respectively, in the Bragg reflection of the inverse spinel structure of magnetite. The observed peak positions of the nanoparticles studied are in agreement with those of the microcrystalline magnetite reference, with some line shifting towards higher angles. This is typical for oxidized magnetite—i.e., maghemite [47]—which has the same crystallographic structure as magnetite. The results obtained show that all the materials studied possess the inverse spinel structure of magnetite or maghemite. However, using only the XRD technique, it is difficult to distinguish between these two compounds as they reveal only a slight difference in their diffraction peak positions [48]. For identification, we used Mössbauer spectroscopy, and the results are described below. It can be easily noticed that peaks at the diffractogram are significantly broadened in comparison with those of microcrystalline magnetite, indicating the nanocrystalline nature of the materials studied. The size of crystallites was determined from the line widths of all identified peaks using Scherrer’s formula, *D = kλ/βcosθ*, where *D* is the average crystallite size, *λ* is the wavelength of X-ray used (1.5406 Å), *k* is a constant (shape factor ~0.9), *θ* is the angle of diffraction, and *β* is the full width at half maximum (FWHM) [49,50].

The values obtained for SPION-CCh, SPION-ACh-Gd, SPION-CCh-B are 10 nm and are 13 nm for SPION-PEG. Taking into account the results obtained from STEM imaging and XRD analysis, it can be concluded that the magnetic cores of all the nanoparticle materials studied are single-crystalline in nature, except for SPION-PEG (with a core size of 30 nm).

#### 3.3.2. Results of Mössbauer Study

**Figure 3:**
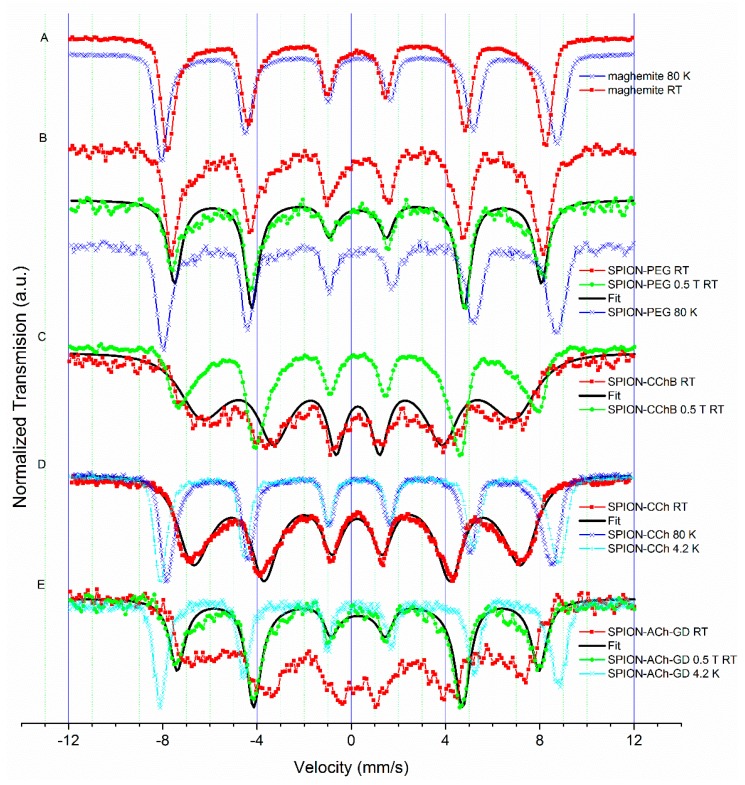
Mössbauer spectra normalized to their most intense lines for all the investigated nanoparticle materials at various temperatures at zero field and in the applied magnetic field (*B* = 0.5 T). Solid black lines are exemplary fits to the measured relaxational spectra. The spectra of microcrystalline maghemite are also shown.

The Mössbauer spectra have been obtained for dried nanoparticle materials at room temperature (RT), 80 K and 4.2 K at zero field and in the applied magnetic field *B* of 0.5 T and are shown in Figure 3.

The Mössbauer spectra of all the SPION samples at room temperature (RT) (Figure 3B–E, red squares) are broadened sextets of much narrower lines at low temperatures, which indicates their relaxational nature, which is characteristic of superparamagnetic materials. The low-temperature spectra closely resemble those of maghemite. The influence of the relaxational character of the magnetic moment of nanoparticle samples is revealed in the broadening of the lines towards the center of the spectrum. For small nanoparticles, their fast relaxation can result in a collapse of the spectrum to a doublet [46]. Fitting the spectra measured at RT to a single relaxational sextet provided the values of their hyperfine parameters (Table 2). The values of the isomer shift (*IS*) obtained, similar for all the samples (0.38 to 0.39 mm/s), are close to those of maghemite and different than those of magnetite [47]. Assuming small or vanishing quadruple splitting (*QS*), such an average *IS* value can be attributed to Fe^3+^ in maghemite at both the octahedral and tetrahedral sites. Taking into account the large half width of the fitted spectra, *G* (0.35 mm/s and more), one cannot exclude the idea that some part of the Fe atoms are of Fe^2.5+^ valence, such as at the octahedral site in magnetite, where fast electron hopping between adjacent sites averages Fe^2+^ and Fe^3+^ states to an intermediate one within the time window of the Mössbauer transition (140 ns). 

The superparamagnetic fluctuation frequency obtained varies between samples, and for SPION-PEG it equals 1.2 MHz and is smaller than the probing frequency of the Mössbauer spectroscopy (1/140 ns = 7 MHz). This result is consistent with the highest blocking temperature, *T_B_* (higher than 310 K), obtained for these nanoparticles from ACMS measurements (see next paragraph). The application of the magnetic field increases the fitted asymmetry parameter *ρ* to 0.98, almost to the static case, where *ρ* = 1. For other SPION samples, the fluctuation frequencies *F* are much higher and more consistent with their *T_B_* lower than 280 K. The unmodified SPION-CCh shows the highest *F* (106 MHz) and modification with gadolinium (SPION-ACh-Gd) or increased shell thickness (SPION-CCh-B) slows the fluctuations down to 43 MHz and 72 MHz, respectively. Application of the external magnetic field slows down the relaxation even more (to about 4 MHz for both modified samples) as expected, because 0.5 T magnetic field induction is far above the saturation value for this material [45]. A high value of the line width, *G*, observed for SPION samples at RT can be attributed to the effect of the size distribution of nanoparticle cores. The change of the resulting line width, *G*, upon the application of the magnetic field is caused by the additional energy introduced to the system. For smaller nanoparticles, this can dominate over the magnetocrystalline anisotropy energy, which decreases with decreasing nanoparticle size [51,52], thus making the frequency distribution narrower. The measured hyperfine field induction (*B_HF_*) was in the range from 44 T to 48 T, being slightly smaller than the average value reported for maghemite (50 T) [53]. This can be explained by the fluctuations decreasing the *B_HF_* values, and their frequency spread can be expected due to the nanoparticle size distribution, as shown in Figure 1. 

The values of the hyperfine parameters obtained by the fitting of the spectra measured at 80 K and at 4.2 K with two sextets are presented in Table 3. Data obtained for samples measured at 4.2 K are close to these determined for the reference maghemite structure. Values of the relative intensity of the tetra- and octahedral components, *IS*, *B_HF_* and *QS*, closely match those of maghemite within the uncertainty margin.

#### 3.3.3. Results of ACMS Measurements

**Figure 4:**
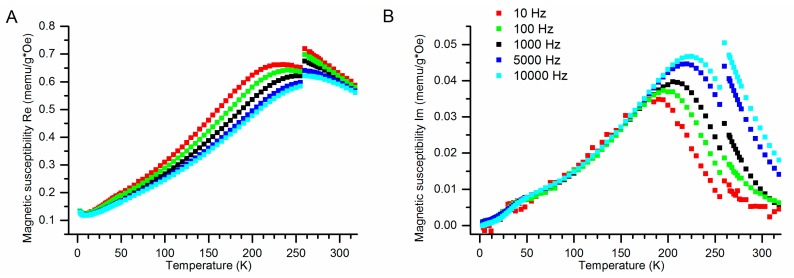
Temperature dependencies of the (**A**) dispersion part of magnetic susceptibility for a water suspension of SPION-CCh-B nanoparticles at various frequencies; (**B**) absorption part of magnetic susceptibility for an aqueous suspension of SPION-CCh-B nanoparticles at various frequencies.

ACMS measurements for all samples were carried out in their water suspension corresponding to approx. a 100 ppm Fe concentration in the 4 K to 323 K temperature range with the Quantum Design Physical Properties Measurement System (PPMS). They have been conducted at frequencies which varied from 10 Hz to 10 kHz, within the exciting field amplitude range from 1 to 10 Oe. The representative results obtained for SPION-CCh-B at various frequencies are presented in Figure 4A for the dispersion part and Figure 4B for the absorption part. One can notice that the maximum of the absorption part of the magnetic susceptibility increases with the increasing frequency of the exciting field and the magnitude of its step at freezing point also increases with frequency. In contrast to that, the overall magnetic susceptibility (dominated by the real part) decreases with the increasing frequency of the exciting field (Figure 4A).

The blocking temperature, *T_B_*, is taken as the maximum of the absorption part (Figure 4B) and is given by the Néel–Arrhenius formula:

(1) $$T_{B}=\frac{KV}{k_{b}\ln\left(\frac{\tau_{m}}{\tau_{0}}\right)}$$

where *K* is the magnetocrystalline anisotropy, *V* is the volume of the particle, *τ*_0_ is the ground state fluctuation time, *τ_m_* is the fluctuation time at a given temperature and *k_b_* is the Boltzmann constant. For all the samples studied, the temperature of this maximum increases with increasing frequency. It reflects the matching of the fluctuation frequencies to the excitation frequency.

**Figure 5:**
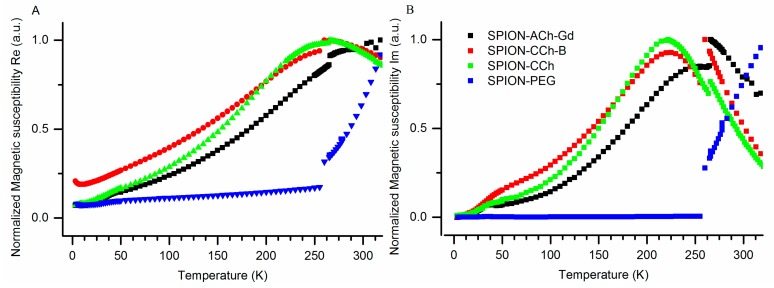
Temperature dependencies of the dispersion part (**A**) and absorption part (**B**) of magnetic susceptibility normalized to their maximum values for aqueous suspensions of the investigated materials measured at 10 kHz.

The drop in magnetic susceptibility observed at freezing temperature, Figure 4 and Figure 5, can be attributed to switching off the movement of the particles. It should be noted that magnetic susceptibility of that kind of material (aqueous suspension) is governed by Brownian [54,55] and Néel’s [56] relaxation processes. The effect observed can be attributed to the suppression of Brown’s relaxation process, which is related mainly to rotational diffusion and occurs when the particle rotates along with its magnetic moment. Thus, the part of the susceptibility remaining below the freezing temperature corresponds to Néel’s process, in which magnetic field repolarizes magnetic moment in the immobilized particle.

In the dispersion part of the magnetic susceptibility measured at 10 kHz (Figure 5A), the temperatures of the maxima differ between the samples, depending on their particle size and composition. The temperatures of the maxima for SPION-ACh-Gd, SPION-CCh-B and SPION-CCh are 294 K, 260 K and 261 K, respectively. The freezing temperature of the suspension is 265 K.

In the absorption part of the magnetic susceptibility measured at 10 kHz (Figure 5B), the temperatures of the maxima are lower than in the dispersion part. The temperatures of maxima for SPION-ACh-Gd, SPION-CCh-B and SPION-CCh are 267 K, 222 K and 222 K, respectively. In both the absorption and the dispersion parts of the SPION-PEG, the temperature of the maximum is above the measurement range.

**Figure 6:**
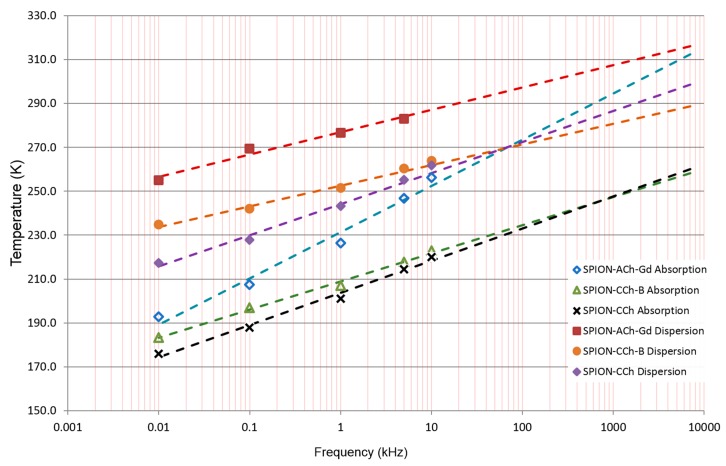
Temperatures at which the susceptibility maximum occurs as a function of the excitation frequency for the absorption (open symbols) and dispersion (full symbols) parts and their linear extrapolation to higher frequencies for an exciting field of 10 Oe.

In general, the transformation of Equation (1) to the frequency domain can be used for the determination of the *KV* product [57]. We used it for a different purpose: namely, we plotted the temperature at which the susceptibility parts have a maximum as a function of the excitation frequency (Figure 6). We observed a linear dependence in the entire frequency range measured. These temperatures are bigger for the dispersion part of all samples. SPION-ACh-Gd exhibits the largest *T_B_* values and the greatest slope. SPION-CCh and SPION-CCh-B are quite similar in their performance, but SPION-CCh-B shows a slightly smaller slope. These linear relations can be extrapolated in order to obtain information regarding what the blocking temperature would be at a given excitation frequency or vice versa.

Considering the possible practical application of the studied nanoparticles as media for hyperthermia, the maximum effect for the material is obtained when the maximum of the absorption part of the susceptibility at a given frequency matches human body temperature. To evaluate this, the temperatures of the maxima versus frequency were extrapolated to human body temperature (see Table 4). For that application, the product of the frequency with the magnetic field strength (here, the exciting field) should be kept below the biological limit (6 MHz Oe) [58]. The results show that SPION-PEG nanoparticles are characterized by the largest absorption, which is related to Brown’s relaxation, arising from the rotation of all particles in the liquid (see below). This is optimal for the use of nanoparticles in magnetic fluid hyperthermia applications [59,60]. Of the SPION materials obtained, SPION-ACh-Gd is the best candidate for this application, but the absorption here is mostly related to Neel’s part. Thus, the material can be considered to be a good candidate for hyperthermia with immobilized nanoparticles, in contrast to SPION-PEG, where the part related to their mobility is dominant, as explained/shown below. It is also worth noting that the difference between SPION-CCh and SPION-CCh-B is of three orders of magnitude.

Magnetic nanoparticles are usually considered as *T*_2_ or *T*_2_* contrast-enhancing agents, where the local field inhomogeneities they introduce and the whole magnetic fluctuation spectrum contribute to this. Regarding their possible *T*_1_ contrast-enhancing effectiveness, the fluctuations should match the Larmor frequency of the scanner (typically from ~10 MHz to 130 MHz). Mössbauer measurements at an external field of 0.5 T indicate that all the SPION samples would be suitable (see Table 2).

**Figure 7:**
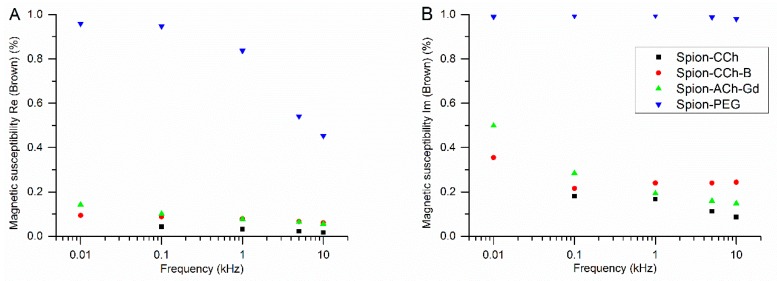
Relative contribution of Brown’s relaxation in (**A**) the dispersion part and (**B**) absorption part of the magnetic susceptibility at freezing temperature as a function of the excitation frequency.

From the height of the steps occurring at freezing temperature in the dispersion and absorption part of the magnetic susceptibility and their total values, Brown’s parts were extracted, and their relative contributions are shown in Figure 7A,B. 

It is worth noting that Brown’s contribution largely dominates in the absorption part for the SPION-PEG material for all the frequencies studied. In the dispersion part, it decreases with increasing frequency. For the SPION materials obtained, Brown’s part makes a minor contribution, but it is generally larger in the absorption part.

## 4. Conclusions

Superparamagnetic iron oxide nanoparticles (SPIONs) coated with various chitosan derivatives and poly(ethylene glycol) were synthesized and characterized. The magnetic core of the nanoparticles was found to contain maghemite, and their hydrodynamic diameters varied from 200 nm for chitosan-coated to 49 nm for PEG-coated particles.

From the fits of the Mössbauer spectra, the superparamagnetic fluctuation frequencies were determined. They extended from tens to hundreds of megahertz at room temperature and were found to decrease in the applied magnetic field of 0.5 T by an order of magnitude. SPION-PEG exhibits a very low fluctuation frequency and is not affected by the applied magnetic field.

The absorption part of the magnetic susceptibility measured in 100 ppm aqueous suspensions of nanoparticles shows maxima in its temperature dependencies in the range located below room temperature. The maxima shift to higher temperatures with increasing excitation frequency, and the extrapolation to room temperature provides frequencies consistent with those obtained from the Mössbauer measurements. The step-like decrease of the magnetic susceptibility was observed at the freezing temperature. From its relative value, Brown’s and Néel’s contributions to the susceptibility were extracted and compared for different nanoparticle cores and coatings. It was found that the PEG coating results in a prevailing Brown’s-like contribution, whereas for chitosan-coated materials, both parts are comparable.

The characteristic frequencies of the superparamagnetic fluctuations observed show that the maximum absorption of the electromagnetic radiation corresponds to a range from tens to hundreds of megahertz; i.e., they can be regarded as suitable for magnetic hyperthermia. In particular, the chitosan-coated SPIONs can be considered as good candidates for the hyperthermia of immobilized nanoparticles, whereas for SPION-PEG, that effect is obtained mainly due to the freely rotating nanoparticles. The SPIONs can also be effective as contrast agents for *T*_1_ weighted scans in MRI.

## Figures and Tables

**Table 1:** The values of the mean hydrodynamic diameter and zeta potential measured by dynamic light scattering (DLS). The uncertainty of zeta potential is also given. SPION: superparamagnetic iron oxide nanoparticles; CCh: cationic derivative of chitosan; ACh: anionic derivative of chitosan; PEG: polyethylene glycol.

Sample Name	Mean Diameter (by Number) *d* [nm]	Zeta Potential *ξ* [mV]
SPION-CCh	121	+32 ± 6
SPION-CCh-B	195	−12 ± 5
SPION-ACh-Gd	93	−34 ± 7
SPION-PEG	49	−9 ± 4

**Table 2:** Hyperfine parameters obtained from the fits of the Mössbauer spectra of nanoparticle materials studied at room temperature.

Fitted Parameters	SPION -PEG RT	SPION -PEG RT; 0.5 T	SPION-CCH RT	SPION-ACh-Gd RT	SPION-Ach-Gd RT; 0.5 T	SPION-CCh-B RT	SPION -CCh-B RT; 0.5 T
*IS* * (mm/s)	0.38(2)	0.39(1)	0.36(1)	0.39(2)	0.4	0.39(1)	0.38(1)
*B_HF_* (T)	48.3(1)	48.3	46.2(2)	44.5(4)	47.7	46(6)	46.5(7)
*G* (mm/s)	0.35	0.35	0.54(1)	0.78(4)	0.32	0.35	0.35
*F* (MHz)	1.2(7)	1.2(4)	106(3)	43(4)	3.8(5)	72(6)	4.1(4)
*ρ*	0.44(4)	0.98(8)	0.91(2)	0.88(5)	0.82(4)	0.78(1)	0.8
*χ^2^*	0.65	1.17	2.19	0.62	1.19	1.33	6.81

* relative to α-Fe.

**Table 3:** Hyperfine parameters for the studied nanoparticles and microcrystalline maghemite obtained from the fit at low temperature (4.2 K and 80 K) of Mössbauer spectra.

Sample	Relative Intensity (%)	*IS* (mm/s)	*B_HF_*(T)	*QS* (mm/s)
SPION-CCh at 4.2 K	61(1)	0.48(1)	52.3(3)	0.02(1)
	39(1)	0.41(1)	50.3(4)	−0.01(1)
SPION-CCh at 80 K	68(1)	0.45(1)	50.9(2)	−0.02(1)
	32(1)	0.39(1)	48.4(5)	−0.02(1)
SPION-ACh-Gd at 4.2 K	64(2)	0.45(1)	52.3(4)	0.01(1)
	36(2)	0.40(1)	50.3(7)	−0.01(1)
SPION-PEG at 80 K	58(3)	0.52(1)	52.3(7)	−0.02(1)
	42(3)	0.46(1)	50.2(9)	−0.02(1)
Maghemite at 80 K	61(1)	0.46(1)	52.5(3)	−0.01(1)
	39(1)	0.40(1)	50.6(5)	−0.02(7)

**Table 4:** Frequency corresponding to the maximum susceptibility extrapolated to 312 K.

Sample	SPION-ACh-Gd	SPION-CCh-B	SPION-CCh
*F_max312_* dispersion (MHz)	2.7	967	30
*F_max312_* absorption (MHz)	6.6	108 000	208
